# Maternal immune activation and antibiotics affect offspring neurodevelopment, behaviour, and microbiome^☆^

**DOI:** 10.1016/j.bbih.2025.101065

**Published:** 2025-07-21

**Authors:** Clara Deady, Jamie FitzGerald, Nirit Kara, Martina Mazzocchi, Adam O'Mahony, Mara Ioana Ionescu, Ruth Shanahan, Saoirse Kelly, Fiona Crispie, Paul D. Cotter, Fergus P. McCarthy, Cathal McCarthy, Gerard W. O'Keeffe, Siobhain M. O'Mahony

**Affiliations:** a Department of Anatomy & Neuroscience, University College Cork, Ireland; b APC Microbiome, University College Cork, Ireland; c Teagasc Food Research Centre, Cork, Ireland; d Department of Physiology - Neuroscience, Carol Davila University of Medicine and Pharmacy, Bucharest, Romania; e Department of Pediatrics, Marie Curie Emergency Children's Hospital, Bucharest, Romania; f Department of Obstetrics & Gynaecology, University College Cork, Ireland; g INFANT Research Centre, Cork, Ireland; h Department of Pharmacology & Therapeutics, University College Cork, Ireland

**Keywords:** Maternal immune activation, Microbiome, Prenatal stress, Inflammation, Neurodevelopment

## Abstract

*In utero* exposure to an increased level of maternal inflammation or a disrupted maternal gut microbiome during pregnancy have been linked to several neurodevelopmental disorders in the offspring. Despite the strong links between these two adverse events, few studies looked at the interaction between the maternal gut microbiome and maternal immune activation (MIA) on the neurodevelopmental outcomes in the offspring. Here, we aim to determine if maternal gut microbiome disruption exacerbated the impact of systemic inflammation on brain development, offspring behaviour, and long-term microbiome changes. A low dose of intraperitoneal lipopolysaccharide (LPS) was administered to pregnant Sprague Dawley rats from gestational day 12–18. Concurrently, an antibiotic cocktail (ampicillin, neomycin, vancomycin) was given in the drinking water to disturb the maternal microbiome. Embryos at gestational day 18 were found to have a reduced body size and weight, along with reduced placental weight following exposure to LPS, with some effects also seen with antibiotic exposure. Offspring exposed to LPS *in utero* were found to have increased anxiety-like behaviours and repetitive-behaviours. No behavioural changes were noted from antibiotic exposure. The expression of 5HT1a receptors in the prefrontal cortex was found to be reduced following LPS exposure. The offspring microbiome varied between the groups, with prenatal antibiotic exposure playing a role in reducing α-diversity and species richness in the periadolescent period. This study highlights the impact of prenatal exposures on different aspects of neurodevelopment. However, additional research is warranted to explore the role of the maternal immune system and microbiome on the offspring development, while also testing the potential therapeutic agents such as probiotics.

## Highlights

• Maternal inflammation and antibiotic use leads to growth restriction in exposed embryos.
• Maternal immune activation leads to increased anxiety-like behaviours in affected offspring.
• Prenatal exposure to maternal antibiotics was found to alter offspring's microbial composition in the periadolescent period.

## 1 Introduction

Prenatal stressors during critical windows of fetal development have deleterious effects on the anatomy and physiology of numerous systems, particularly the nervous system (Minakova and Warner, 2018; Van Den Bergh et al., 2020; Yeramilli et al., 2023). The outcome of this *in utero* exposure may be associated with increased risks of developing neurodevelopmental disorders, such as autism spectrum disorder, attention deficit hyperactivity disorder, and schizophrenia, amongst other neuropsychiatric conditions (Beversdorf et al., 2018; Jallow et al., 2024; Lipner et al., 2019). The activation of the maternal immune system due to an infection during pregnancy is one particular prenatal stressor that has been associated with unfavourable neurodevelopmental outcomes (Vitor-Vieira et al., 2024; Xuan and Hampson, 2014; Zuckerman and Weiner, 2005). This connection has been consolidated by examining numerous global crises such as the rubella epidemic of 1964, the influenza pandemic of 1918, and more recently, the COVID-19 pandemic of 2020, all of which have been associated with an increased risk of a range of neurodevelopmental disorders (Brown, 2006; Brown et al., 2000, 2001; Dubey et al., 2022; Kępińska et al., 2020; Shook et al., 2022). The evidence from these diverse events suggests that it is not a specific set of pathogens that lead to these neurodevelopmental disorders, but rather that they may be linked via excessive and aberrant maternal immune activation. This has been shown through numerous rodent models which illicit similar neurobehavioural outcomes despite immune stimulation with different mimetics (Solek et al., 2018).

Administration of the viral RNA polyinosinic:polycytidylic acid (Poly(I:C)) or the bacterial cell wall endotoxin lipopolysaccharide (LPS) illicit strong immune responses in rodents (Bao et al., 2022; Bucknor et al., 2022). These models have been shown to result in altered levels of fundamental neurotransmitters such as serotonin and dopamine, disruption in the neuroanatomical architecture, and increased microglia activation. These changes may result in severe behavioural changes and lead to the development of anxiety-like and autistic-like phenotypes (Aria et al., 2020; Estes and McAllister, 2016; Murray et al., 2019). Through studying the cellular and functional changes, numerous drivers have been postulated. These include abnormal microglia activation affecting various cellular functions such as pruning and apoptosis, excessive generation of pro-inflammatory cytokines, or the hypermethylation of certain synaptic promoters (Labouesse et al., 2015; Solek et al., 2018).

Building upon this, the maternal gut microbiome has recently been identified as a key player in the development of the fetus (Sharon et al., 2016; Warner, 2019). The gut microbiome can be influenced directly by poor diet, medication use, and stress (Cryan et al., 2019). The gut-brain axis is defined as a bidirectional pathway of communication between the brain and the gut, with one being able to modify the other, positively or negatively, through vagus nerve activity, alterations in neurotransmitters, activation of the immune system, and certain bacterial metabolites (Cryan et al., 2019). Through this pathway, it is possible that prenatal stressors, such as maternal immune activation (MIA), may disrupt the maternal gut subsequently causing neurodevelopmental complications and potentially having a cumulative effect.

Most of this work has been performed in animal models, examining the effect of microbial depletion or germ-free conditions on neurodevelopment. Germ-free rodents, which simply lack a gut microbiome, have been shown to display altered anxiety and social behaviours, reductions in choroid plexus integrity, and disrupted myelination (Forssberg, 2019; Hoban et al., 2016; Knox et al., 2023). Alterations in numerous transcriptional pathways that are involved in neuronal plasticity and neurotransmission have also been reported (Stilling et al., 2015). Through microbial depletion studies, a less drastic and more translatable model, it has been shown that suboptimal support from the maternal microbiome can lead to severe fetal neurodevelopmental complications that range from distorted tactile sensitivity to altered sociability levels in the offspring (Hassib et al., 2023; Vuong et al., 2020).

Antibiotic use during pregnancy is both common and often necessary to fight infections (Bookstaver et al., 2015; Nakitanda et al., 2024). While most antibiotics are accepted as safe, other classes such as streptomycin or tetracycline may induce teratogenic effects (Bookstaver et al., 2015; Norwitz and Greenberg, 2009). Additionally, frequent antibiotic use can disrupt the microbiome through reduction in the beneficial microbial species, diversity, and the promotion of overgrowth of unfavourable species, which is postulated to lead to neurodevelopmental changes in animal models (Becattini et al., 2016; Ferrer et al., 2017).

In this study we combined an LPS model of MIA and maternal gut dysbiosis to mimic a maternal infection that would require antibiotic treatment. We aimed to investigate the behaviour of the offspring following their exposure to the combination of prenatal stressors, with a specific focus on anxiety and autistic-like phenotypes, while also examining the impact on relevant brain areas and the preadolescent microbiome.

## 2 Methods

### 2.1 Animals and housing

Male and female Sprague Dawley rats were purchased from Envigo, United Kingdom aged approximately 7 weeks old and housed in the Biological Services Unit, Western Gateway Building, University College Cork. All rats had water and standard chow *ad libitum*. Rats were housed in RC2F type cages (1575 cm^2^) with sawdust bedding and cardboard tubes. All cages were held in a controlled environment, air temperature set to 21 °C ± 1 °C, and a 12-hour light/dark cycle with light between 7am and 7pm. Rats were allowed to habituate for 10 days before they were moved to a smaller breeding cage (960 cm^2^). Embryonic day (E) 0 was defined upon identification of the vaginal plug. All procedures were conducted with approval from the Animal Experimentation Ethics Committee at University College Cork and the Health Products Regulatory Authority, in accordance with the recommendations of the European Directive 2010/63/EU.

### 2.2 Study design

The dams were randomly assigned to either cohort 1 or cohort 2. Both cohorts received identical treatment as discussed in section 2.3. However, rats from cohort 1 were culled via decapitation on E18 and the embryos were then assessed, as described in section 2.4. For cohort 1, there were 2 dams in the antibiotic (ABX) group, 3 dams in both the saline and the combined group (LPS/ABX), and 4 for the LPS group. For cohort 2, each group had 6 dams, except for saline which had 5. On E18, the dams from cohort 2 were housed individually in small cages (960 cm^2^) and allowed to give birth. Day of birth was assigned as postnatal day (P) 0. Pups stayed with their mothers until P21 when they were weaned and housed 2 males or 2 females per small cages (960 cm^2^). This produced a total of 20 offspring for the saline group, 23 offspring for the LPS/ABX group due to a single female pup born to one dam, and 24 offspring for both the LPS and the ABX groups. Dams were culled on P21.

### 2.3 Maternal immune activation and gut dysbiosis model

On E12, pregnant females were randomly assigned to one of 4 groups: saline, ABX, LPS, LPS/ABX. Dams were separated into cages based on their treatment group, with at least two dams in each cage. Those in the saline group received 0.5 ml of saline intraperitoneally with a 25G needle from E12-18, alternating the injection site from right to left, every other day. Those in the ABX group received 1 mg/ml of neomycin (Neomycin sulfate 1405-10-3, Discovery Fine Chemicals), 1 mg/ml of ampicillin (Ampicillin Sodium Salt 69-52-3, Discovery Fine Chemicals), and 0.5 mg/ml of vancomycin (Vancomycin hydrochloride 1404-93-9, Discovery Fine Chemicals) dissolved in drinking water. The ABX water was changed every 3 days and was given on E12-18. Rats were weighed daily each morning. Dams received 50 μg/kg of LPS (Lipopolysaccharides from *Escherichia coli* O26:B6, Merck) dissolved in 0.5 ml saline intraperitoneally using a 25G needle from E12-18, alternating the injection site from right to left, every other day. Those in the LPS/ABX group received both LPS and antibiotics from E12-18.

### 2.4 Assessment of the embryos

Cohort 1 of the treated dams was taken on E18 for morphological assessment of the embryos. Dams were anesthetised using isoflurane (Vetflurane® 1000 mg/g), pedal, tail, and palpebral reflexes checked, and decapitated. Embryos were removed via laparotomy and placed into phosphate buffer saline (PBS). The weight and crown to rump length of each embryo was recorded, along with its placental weight.

### 2.5 Ultrasonic vocalisation recordings

On P9, the pups underwent ultrasonic recordings to measure their affective state. Pups were removed from their home cage one by one. Pups were placed in a small grey box which was placed under a styrofoam box that was used to insulate the calls and block out external noise. The microphone was threaded through the styrofoam box and hung approximately 10 cm above the pup. The pups were recorded for 3 min before being returned to their home cage and gently rolled in the bedding before being placed in the nest once again. Ultrasonic vocalisations were recorded and analysed using UltraVox XT v3.2.

### 2.6 Open field test

On P26, the Open-Field test was performed to measure anxiety-like behaviour and locomotion. The pups were allowed to habituate to a separate room for 45 min before the beginning of the test. The circular open-field test (90 cm diameter) was situated in a dimmed room with the arena being brightly lit with lamps (1000 lux) to induce anxiety. The rats were allowed to explore the arena for 20 min. The rat was then removed and returned to its home cage and the arena was wiped down with 70% ethanol to remove any olfactory cues before the next rat entered. The total distance travelled, mean speed, number of immobile episodes (a stationary period for >5 s), and time spent in the centre of the arena was scored using ANY-Maze v7.16.

### 2.7 Marble burying

On P33, the pups underwent Marble Burying to measure autistic-like and anxiety-like behaviours. The rats were allowed to habituate to a separate room for 30 min. Following the habituation period, rats were placed into small cages (960 cm^2^) with black marbles (15.9 mm diameter) arranged in a 4 x 5 arrangement on top of 5 cm of tightly packed sawdust bedding. The rats were allowed explore for 30 min before being removed from the test arena and returned to their home cage. The number of buried marbles (2/3 of the marble underneath the bedding) were then counted.

### 2.8 3-Chamber Social test

On P40, the 3-Chamber Social test was performed to measure sociability and social novelty. Rats were allowed to habituate in a dimmed room for 45 min before beginning the test. The rectangular arena (57 cm × 99 cm) was divided into 3 separate chambers, connected by a small open doorway so the rat could explore all 3 chambers. The two outer chambers contained a small container to hold the non-test rats. In the first 10 min, the experimental rat was allowed to explore the whole arena. This is known as the habituation phase. The next 10 min, a novel, age- and sex-matched conspecific rat was included in one of the containers, with the other container empty. The time the experimental animal spent with conspecific rat was recorded and defined as sociability. In the last 10 min, another novel, age- and sex-matched conspecific was included in the remaining container in the other chamber The rat used in the sociability phase was left in its container. The experimental animal was allowed to interact with both conspecifics and the time spent with the new rat was measured and compared to the time spent with the now ‘familiar’ rat and defined as social novelty. Following the test, all rats were returned to their home cage and the arena was wiped down with 70% ethanol to remove any olfactory cues. Sociability and social novelty were scored using BORIS v7.13.9.

### 2.9 Transcardial perfusions

On P47, the rats were euthanised by intraperitoneal injection with a 25G needle with 0.8 ml/kg of 200 mg sodium pentobarbital (Euthatol). The palpebral, tail, and pedal reflexes were checked before beginning perfusion. The animal was secured to the dissection board and the thoracic cavity was opened. With the heart still beating, a 15G cannula was inserted into the left ventricle. An incision was made in the right ventricle to allow blood to drain. Cold 10 mM PBS was pumped through the body using a Watson Marlow 101 U/R peristaltic pump until the perfusate ran clear. Following this, the pump was switched to 4 % paraformaldehyde (PFA). Following the blanching of the liver and feet, stiffness of the neck, and fixation tremors, the rat was decapitated and the whole brain extracted.

### 2.10 Tissue preparation

Following the brain removal, it was immersed in 4% PFA and stored at 4 °C overnight. The following day, the brain tissue was transferred to 30% sucrose and kept at 4 °C until fully submerged, then snap-frozen in liquid nitrogen, for long-term storage at −80 °C. 30 μm sections were cut using a freezing stage cryostat (Leica CM1950) and sections were kept in cryoprotectant (10 mM PBS with 30% ethylene glycol, 25% glycerol, 20% water) until mounting on Superfrost™ Plus Adhesion Microscope Slides and kept at −20 °C until used for immunohistochemistry.

### 2.11 5HT1a receptor staining

The amygdala and prefrontal cortex were stained for 5HT1a receptor expression. Sections were removed from the freezer and brought to room temperature. Sections were washed 3 times, 10 min each using TXTBS (Triton-X tris-buffered saline). Non-specific binding was then blocked using 10 % goat serum in Tris-buffered saline (TBS) for 1 h. Following this, primary antibody (anti-5HT1a receptor, Merck AB15350) (1:1000 dilution) and 1% goat serum was diluted in TBS. 200 μL was added to each slide and allowed to incubate overnight at 4 °C. Sections were washed 3 times, 10 min each with TXTBS. The secondary antibody (Alexa Flour 488 conjugated secondary antibody, Invitrogen) (1:500 dilution) was diluted in TBS with 1% goat serum and 200 μL was added to each slide. Following a 2-h incubation at room temperature in the dark, slides were washed 3 times, 10 min each with TXTBS. Nuclei were stained with DAPI (4′-6-Diamidino-2-phenylindole, Sigma) (1:3000 dilution) in TBS for 5 min in the dark at room temperature. Finally, slides were washed 3 times, 10 min each with TBS before leaving to dry and cover-slipping with DAKO Fluorescence Mounting Medium. Slides were stored in the dark at 4 °C until imaged on the Olympus BX53 Upright Microscope. Intensity of receptor expression was measured using ImageJ software.

### 2.12 DNA extraction

Faecal pellets were collected on P40 and DNA was extracted using QIAamp DNA Micro Kit (Qiagen) as per the manufacturer's instructions Quantification of DNA was assessed using ND-1000 Spectrophotometer (NanoDrop). Extracted DNA was stored at −80 °C until sent for sequencing.

### 2.13 DNA sequencing

DNA was standardised to 5 ng/μL and the 16S library was prepared using a fivefold miniaturized version of the Illumina 16S metagenomics library preparation protocol on the Echo 525 (Labcyte). The initial PCR was carried out using 0.5 μl DNA (at 5 ng/μL), 1 μL of both the forward and reverse primers (Illumina 16S metagenomics library preparation protocol) at 1 μM, and 2.5 μL 2 x Kapa Hifi Hotstart Ready mix. DNA and reagents were added to 96-well plates using the Echo 525 (Labcyte) and spun briefly to mix. The PCR conditions followed the Illumina 16S metagenomics protocol. Following this, the plates were spun, and 15 μl molecular grade water was added to each sample. Samples then underwent Ampure (Beckman Coulter) purification on the Beckman i7 (Beckman Coulter), the second PCR was set up again using the Echo 525 to transfer the liquid volumes to fresh plates. The reactions were again miniaturized once again to have a final volume of 5 μl 2x Kapa Hifi Hotstart Ready mix, 1 μl purified PCR product, 1 μl N7 index primer (Illumina), 1 μl S5 index primer (Illumina), and 2 μl molecular grade water. The plate was then spun, and index PCR performed as detailed in the Illumina 16S metagenomics protocol. No template controls were included in the initial PCRs and carried through all steps of library preparation. Samples were quantified using a Qubit High Sensitivity Assay and pooled equimolarly. An aliquot of the pool was then cleaned using a 0.8 X ratio of Ampure (Beckman Coulter) beads. The final pool was measured using Qubit and assessed on an Agilent high sensitivity chip (Agilent). The pool was diluted to 1 nM and mixed with 1 nM PhiX (Illumina) at 40% (v/v). The mixture was then loaded onto a P1 600 cycle cartridge and sequenced on the NextSeq 2000 following Illumina guidelines.

### 2.14 Bioinformatics

Illumina sequence data was retrieved via the *basespace* CLI (Illumina, USA). Sequence quality was evaluated using *FastQC* and *MultiQC* (Ewels et al., 2016) (Babraham Bioinformatics - FastQC A Quality Control tool for High Throughput Sequence Data), and synthetic sequences were removed using *Trimmomatic* (Bolger et al., 2014) (sliding filter window 6:15; minlength 299). *FIGARO* (FIGARO: An efficient and objective tool for optimizing microbiome rRNA gene trimming parameters | bioRxiv) was used to find optimal *dada2::filterAndTrim* parameters for each sample, which were then averaged to a single trimming value across the study (3′ trim: 270,214; maxEE: 1,2; 5’ trim: 0,0). Further *DADA2* (Callahan et al., 2016) parameters were set to determine and quantify unique amplicon sequence variants (ASVs) across the study: *learnErrors*: randomise = TRUE, nbases = 1 × 10^9^; *mergePairs*: minimum overlap of 25bp, maximum allowed mismatch of 0bp; *AssignTaxa*: RBD used the “consensus” method. Other parameters remained at default values. *DADA2* (assignSpecies) and DECIPHER (Wright, 2016) were used to generate taxonomic assignments from the Silva 138 release (Quast et al., 2013). *Decontam* (Davis et al., 2018) was used to detect contaminant ASVs based on prevalence between normal and control samples. Bimeras, contaminants, ASVs of unknown phylum, and ASVs outside of the expected length (397–428bp) were checked before being removed.

### 2.15 Statistics

As behavioural readouts were the primary outcome of this study, all power calculations were determined based on the marble burying test. Using GPower 3.1, to reach statistical significance, a total of 6 dams per group were required, with 12 males and 12 females per group to allow us to see sex differences within the group. Power calculations were approved by a biostatistician, Department of Mathematics, University College Cork.

**Fig. 1:**
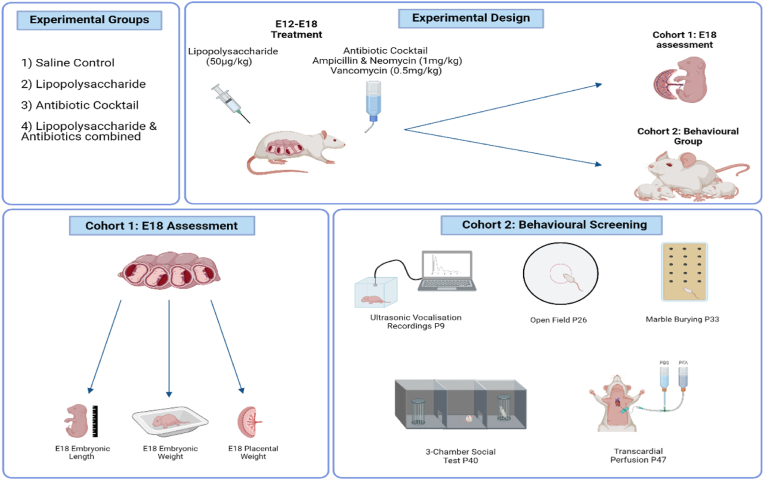
**Study design detailing experimental groups, maternal treatments, and the fate of cohort 1 and 2.** Abbreviations: E: Embryonic day; P: Postnatal day.

**Fig. 2:**
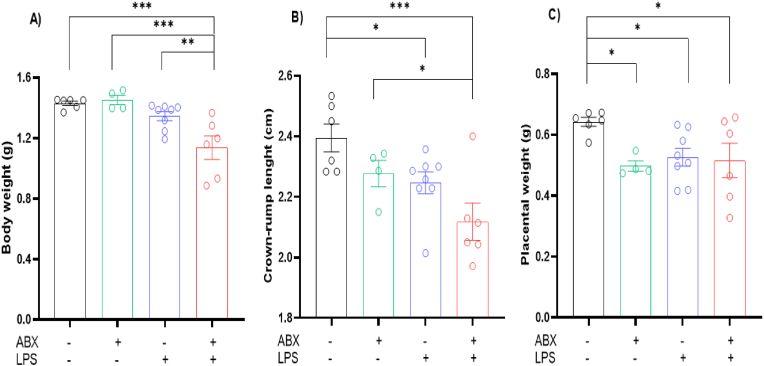
**Significant morphological changes seen in E18 following inflammation and microbial disruption.** A) Pup weight on embryonic day 18. B) Pup crown-rump length on embryonic day 18. C) Placental weight on embryonic day 18. N = 2–4 dams per group. 1 male and 1 female, 2 data points averaged to give 1 data point per sex per litter. ∗ = P=<0.05, ∗∗ = P=<0.01, ∗∗∗ = P=<0.001.

**Fig. 3:**
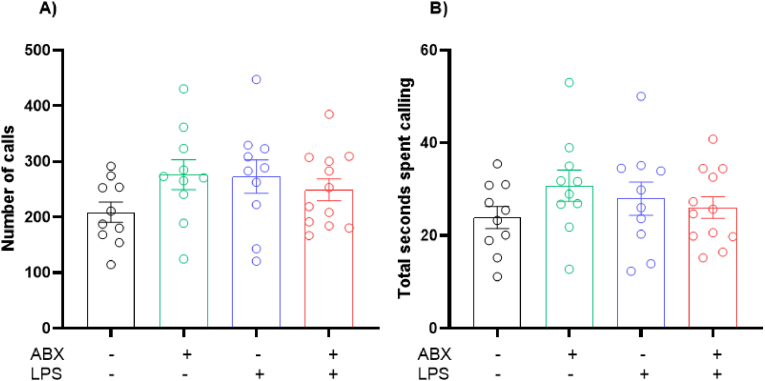
**No significant changes seen in ultrasonic vocalisations following inflammation and microbial disruption.** A) Number of ultrasonic calls recorded within a 3-min period. B) Total time spent calling within a 3-min period. Recordings taken on postnatal day 9. N = 5–6 dams per group. 1 male and 1 female, 2 data points averaged to give 1 data point per sex per litter.

**Fig. 4:**
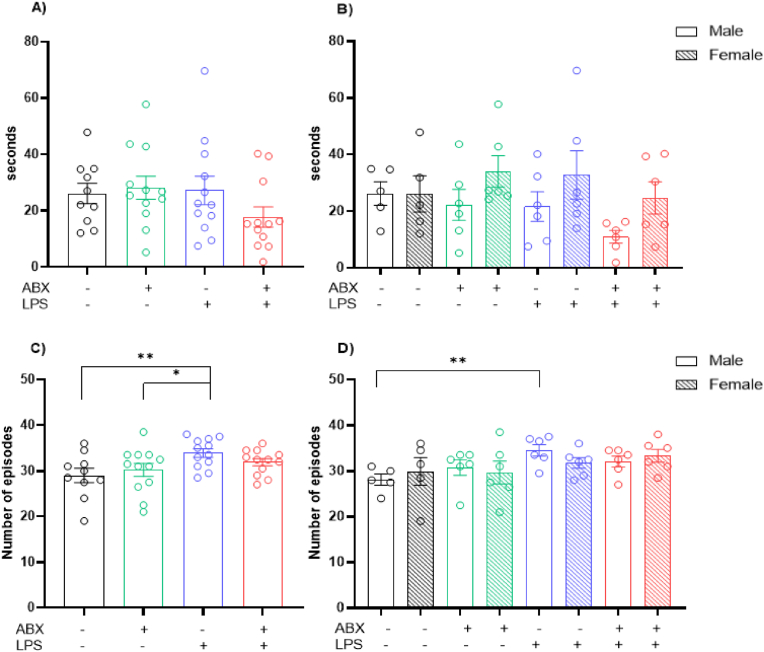
**Increased anxiety following prenatal inflammation exposure.** A) Time spent in the centre within a 20-min period. B) Sex differences in time spent in the centre within a 20-min period. C) Number of immobile episodes (>5 stationary seconds) within a 20-min period. D) Sex differences in the number of immobile episodes (>5 stationary seconds) within a 20-min period. Behaviour performed on postnatal day 25. N = 5–6 dams per group. 1 male and 1 female, 2 data points averaged to give 1 data point per sex per litter. ∗ = P=<0.05, ∗∗ = P=<0.01.

**Fig. 5:**
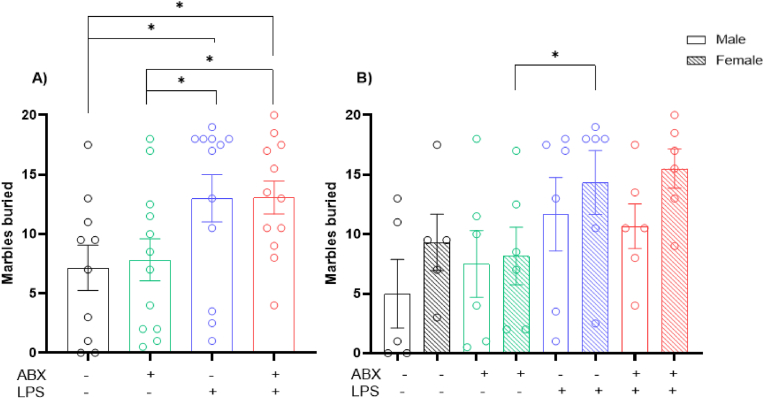
**Increased repetitive behaviours following prenatal inflammation.** A) Number of marbles buried within a 30-min period. B) Sex differences in number of marbles buried within a 30-min period. Behaviour performed on postnatal day 33. N = 5–6 dams per group. 1 male and 1 female, 2 data points averaged to give 1 data point per sex per litter. ∗ = P=<0.05.

**Fig. 6:**
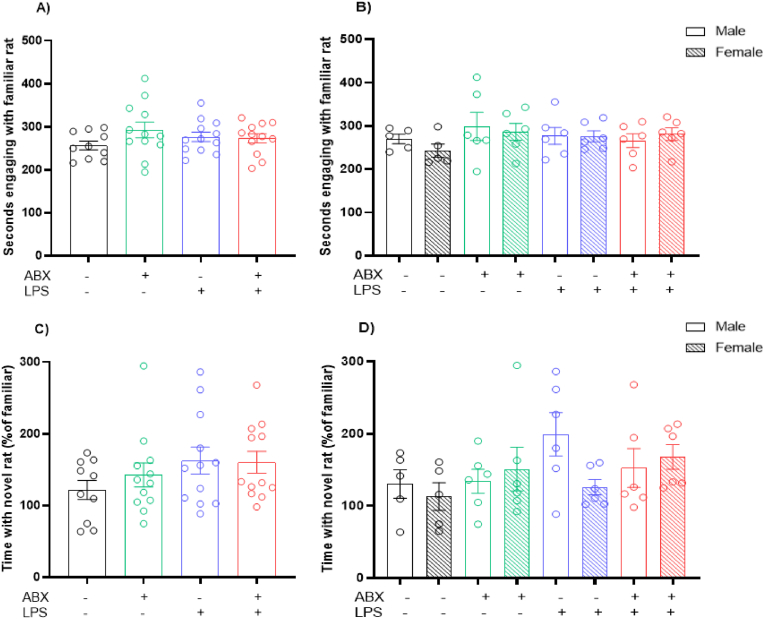
**No changes to sociability following prenatal exposures.** A) Time spent with a novel, conspecific rat across a 10-min period. B) Sex differences in time spent with a novel, conspecific rat across a 10-min period. C) Time spent with a novel, conspecific rat across a 10-min period expressed as a percentage of time spent with a familiar conspecific. D) Sex differences in time spent with a novel, conspecific rat across a 10-min period expressed as a percentage of time spent with a familiar conspecific. Behaviour performed on postnatal day 40. N = 5–6 dams per group. 1 male and 1 female, 2 data points averaged to give 1 data point per sex per litter.

**Fig. 7:**
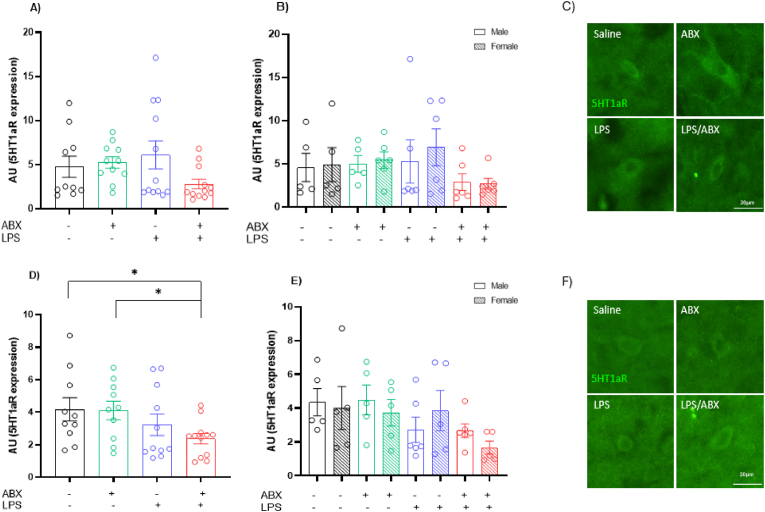
**Reduction in 5HT1a receptor expression in the prefrontal cortex following in utero exposure to inflammation.** A) 5HT1aR expression in the amygdala. B) Sex differences in 5HT1aR expression in the amygdala. 5HT1aR expression. C) Representative photomicrographs on 5HT1aR expression in the amygdala. D) 5HT1aR expression in the prefrontal cortex. E) Sex differences in 5HT1aR expression seen in the prefrontal cortex. Significant in males with two-way ANOVA only. F) Representative photomicrographs on 5HT1aR staining in the prefrontal cortex. N = 5–6 dams per group, 1 male and 1 female per litter. ∗ = P=<0.05.

For Fig. 2, Fig. 3, Fig. 4, Fig. 5, Fig. 6, Fig. 7, statistical analysis was performed using GraphPad Prism v8.3.0. All analysis were carried out using a 2-way ANOVA with *post hoc* Fisher's test where appropriate. Results were deemed significant when p < 0.05. All data presented as mean ± SEM. For Fig. 2, Fig. 3, Fig. 4, Fig. 5, Fig. 6, 2 data points for both males and females were averaged to give 1 data point per sex per litter. For Fig. 7, Fig. 1 male and 1 female was used per litter.

For microbiome analysis, for visualisation purposes only, ASVs below 1 % abundance and/or prevalence in ≥9 samples were combined to “other”. Remaining taxa were summed across different taxonomic levels (phylum, family, genus). Diversity analyses (α, β) were carried out using vegan (R - Vegan Tutorial,)to compute Shannon's H, Inverse Simpson's index, Chao1 richness, Bray-Curtis dissimilarity for PERMANOVA, the Hellinger transformation of abundances for constrained correspondence analysis, and significance of community structure (*vegan::betadisper, ::adonis2, ::anova*). For differential analysis, ASV count data was transformed using count-zero mutiplicative replacement of zeros with *zCompositions::cmultrepl* (Palarea-Albaladejo and Martín-Fernández, 2015) to allow a centre log-ratio transform.

## 3 Results

### 3.1 Inflammation and microbiome disruption during pregnancy reduces pup size and placental weight

Two-way ANOVA analysis of E18 body weight revealed significant interaction and LPS effects (Interaction Effect: P = 0.0242, F(1, 20) = 5.942; ABX Effect: P = 0.0617, F(1, 20) = 3.918; LPS Effect: P = 0.0004, F(1, 20) = 18.05). LSD Fisher's *post hoc* test noted numerous differences between *in utero* exposures. Body weight reduction was seen in LPS/ABX group compared to the saline control group (P = 0.0002), as well as the LPS group (P = 0.0026) and the ABX group (0.0003) (Fig. 2A). Overall crown-rump length did not show an interaction effect, but a two-way ANOVA noted a strong ABX and LPS effect (Interaction Effect: P = 0.9171, F(1, 20) = 0.0111; ABX Effect: P = 0.0207, F(1, 20) = 6.310; LPS Effect: P = 0.0053, F(1, 20) = 9.796). *Post hoc* testing revealed a decrease in crown-rump length in the LPS/ABX group in comparison to the ABX group (P = 0.0477). This reduction in crown-rump length was also evident in the LPS group (P = 0.0287) and the LPS/ABX group (P = 0.0005) compared to the saline group (Fig. 2B).

Two-way ANOVA also revealed an ABX effect on placental weight, but no interaction or LPS effect (Interaction Effect: P = 0.0856, F(1, 20) = 3.270; ABX Effect: P = 0.0489, F(1, 20) = 4.399; LPS Effect: P = 0.2019, F(1, 20) = 1.741). LSD Fisher's *post hoc* revealed a significant reduction in placental weight in the ABX group (P = 0.0243), the LPS group (P = 0.0192), and the LPS/ABX group (P = 0.217) in comparison to the saline control group (Fig. 2C).

### 3.2 Inflammation and maternal microbiome disruption during pregnancy does not alter the frequency or length of pup ultrasonic vocalisations

Two-way ANOVA revealed no significant changes seen in the number of calls (Interaction Effect: P = 0.0629, F(1, 38) = 3.672; ABX Effect: P = 0.3648, F(1, 38) = 0.8413; LPS Effect: P = 0.4472, F(1, 38) = 0.5898) (Fig. 3A). Furthermore, no significant changes were seen in the total call duration across the 3-min test period as seen with a two-way ANOVA (Interaction Effect: P = 0.1390, F(1, 38) = 2.284; ABX Effect: P = 0.4062, F(1, 38) = 0.7055; LPS Effect: P = 0.9124, F(1, 38) = 0.0122) (Fig. 3B).

### 3.3 Rats show anxiety-like behaviour due to inflammation and maternal microbiome disruption *in utero*

Time spent in the centre of the arena, used as an indication of anxiety-like behaviour, was not statistically significant following a two-way ANOVA (Interaction Effect: P = 0.1802, F(1, 42) = 1.858; ABX Effect: P = 0.3804, F(1, 42) = 0.7857; LPS Effect: P = 0.2754, F(1, 42) = 1.221 (Fig. 4A). No significant differences were seen when examining the sexes either (Male: Interaction Effect: P = 0.4660, F(1, 19) = 0.5535; ABX Effect: P = 0.1193, F(1, 19) = 2.661; LPS Effect: P = 0.0944, F(1, 19) = 3.099) (Female: Interaction Effect: P = 0.2426, F(1, 19) = 1.455; ABX Effect: P = 0.9905, F(1, 19) = 0.0001; LPS Effect: P = 0.8446, F(1, 19) = 0.0394) (Fig. 4B).

Two-way ANOVA found a significant LPS effect in the number of immobile episodes, a measure of anxiety-like behaviour (Interaction Effect: P = 0.1880, F(1, 42) = 1.791; ABX Effect: P = 0.7429, F(1, 42) = 0.1091; LPS Effect: P = 0.0084, F(1, 42) = 7.638) (Fig. 4C). LSD Fisher's *post hoc* test revealed a significant increase in the number of immobile episodes in the LPS group in comparison to both the saline group (P = 0.007) and the ABX group (P = 0.303). Finally, males displayed a significant LPS effect in number of immobile episodes following two-way ANOVA analysis (Interaction Effect: P = 0.0814, F(1, 19) = 3.386; ABX Effect: P = 0.9334, F(1, 19) = 0.0071; LPS Effect: P = 0.0122, F(1, 19) = 7.888) (Fig. 4D). LSD Fisher's *post hoc* test revealed a significant increase in the number of immobile episodes in the LPS group in comparison to the saline group (P = 0.0046). No significant changes were seen in females (Interaction Effect: P = 0.6666, F(1, 19) = 0.1915; ABX Effect: P = 0.7486, F(1, 19) = 0.1058; LPS Effect: P = 0.1996, F(1, 19) = 1.766) (Fig. 4D).

### 3.4 Rats show stereotypical, repetitive behaviours due to inflammation *in utero*

Two-way ANOVA revealed statistically significant changes with an LPS effect in the number of marbles buried (Interaction Effect: P = 0.8667, F(1, 42) = 0.0285; ABX Effect: P = 0.8302, F(1, 42) = 0.0465; LPS Effect: P = 0.0032, F(1, 42) = 9.755) (Fig. 5A). LSD Fisher's *post hoc* test revealed a significant increase in the number of marbles buried in both the LPS group (P = 0.0281) and combined LPS/ABX group (P = 0.0261) compared to the saline control group. An increase in burying was also found between both the LPS group (P = 0.0412) and the combined LPS/ABX group (P = 0.0381) compared to the ABX group. Regarding the males, no main effect was seen with a two-way ANOVA (Interaction Effect: P = 0.5251, F(1, 19) = 0.4192; ABX Effect: P = 0.7844, F(1, 19) = 0.077; LPS Effect: P = 0.0847, F(1, 19) = 3.309) (Fig. 5B). Females displayed a strong LPS effect (Interaction Effect: P = 0.6255, F(1, 19) = 0.2461; ABX Effect: P = 0.9943, F(1, 19) = 5.169e-005; LPS Effect: P = 0.0152, F(1, 19) = 7.115) (Fig. 5B). LSD Fisher's *post hoc* test noted an increase in buried marbles in the LPS/ABX group (P = 0.0271) in comparison to the ABX group.

### 3.5 Rats do not show altered sociability due to inflammation or maternal microbiome disruption exposure *in utero*

Two-way ANOVA revealed no significant effect of *in utero* exposures on sociability (Interaction Effect: P = 0.1427, F(1, 42) = 2.231; ABX Effect: P = 0.2235, F(1, 42) = 1.527; LPS Effect: P = 0.9814, F(1, 42) = 0.0005) (Fig. 6A). No significant differences were seen within the sexes either (Male: Interaction Effect: P = 0.3820, F(1, 19) = 0.8009; ABX Effect: P = 0.7015, F(1, 19) = 0.1514; LPS Effect: P = 0.5630, F(1, 19) = 0.3465) (Female: Interaction Effect: P = 0.2377, F(1, 19) = 1.487; ABX Effect: P = 0.1422, F(1, 19) = 2.345; LPS Effect: P = 0.3903, F(1, 19) = 0.7728) (Fig. 6B). Regarding social novelty, a two-way ANOVA detected no main effects (Interaction Effect: P = 0.4831, F(1, 42) = 0.5007; ABX Effect: P = 0.5745, F(1, 42) = 0.3201; LPS Effect: P = 0.0833, F(1, 42) = 3.146) (Fig. 6C). When examining differences within the sexes, no significant alterations were observed (Male: Interaction Effect: P = 0.3148, F(1, 19) = 1.066; ABX Effect: P = 0.3918, F(1, 19) = 0.7680; LPS Effect: P = 0.0903, F(1, 19) = 3.186) (Female: Interaction Effect: P = 0.9282, F(1, 19) = 0.0083; ABX Effect: P = 0.0696, F(1, 19) = 3.698; LPS Effect: P = 0.4844, F(1, 19) = 0.5086) (Fig. 6D).

### 3.6 Inflammation reduces 5HT1a receptor expression in the prefrontal cortex, but not in the amygdala

The intensity of 5HT1a receptor expression in the amygdala was analysed via a two-way ANOVA and revealed no significant changes (Interaction Effect: P = 0.0900, F(1, 41) = 3.015; ABX Effect: P = 0.2037, F(1, 41) = 1.669; LPS Effect: P = 0.6197, F(1, 41) = 0.2501) (Fig. 7A). No significant changes were seen between males or females (Male: Interaction Effect: P = 0.4224, F(1, 18) = 0.6741; ABX Effect: P = 0.5706, F(1, 18) = 0.3338; LPS Effect: P = 0.6868, F(1, 18) = 0.1679) (Female: Interaction Effect: P = 0.1362, F(1, 19) = 2.421; ABX Effect: P = 0.2385, F(1, 19) = 1.481; LPS Effect: P = 0.8091, F(1, 19) = 0.06004) (Fig. 7B). The prefrontal cortex revealed a significant reduction in 5HT1a receptor expression with an LPS effect (Interaction Effect: P = 0.4946, F(1, 39) = 0.4754; ABX Effect: P = 0.4190, F(1, 39) = 0.6670; LPS Effect: P = 0.0248, F(1, 39) = 5.454) (Fig. 7D). LSD Fisher's *post hoc* test noted a reduction in receptor expression in the LPS/ABX group in comparison to the ABX group (P = 0.0369) and the saline group (P = 0.03). While no differences were seen between females, an LPS effect was found in males following two-way ANOVA analysis (Male: Interaction Effect: P = 0.8994, F(1, 18) = 0.0167; ABX Effect: P = 0.9513, F(1, 18) = 0.0038; LPS Effect: P = 0.0269, F(1, 18) = 5.808) (Female: Interaction Effect: P = 0.3437, F(1, 16) = 0.9522; ABX Effect: P = 0.224, F(1, 16) = 1.60; LPS Effect: P = 0.2726, F(1, 16) = 1.291) (Fig. 7E).

### 3.7 Exposure to inflammation and maternal microbiome disruption *in utero* reduces diversity and alters the gut microbiome

**Fig. 8:**
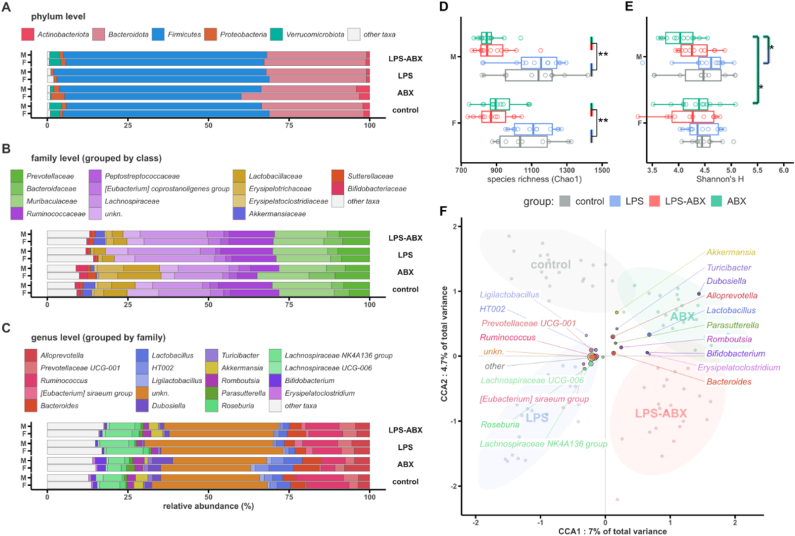
Microbiome disruption and inflammation lead to enduring changes in the microbiome of *offspring.* A-C) Mean relative abundances of the pup microbiome at postnatal day 40, summarised at phylum, family and genus levels, grouped by group and sex. Taxa at family level share a colour if within the same class, while taxa at genus level share a colour if within the same family. Taxa not present above 1% in 9 or more samples are grouped to “other taxa”. D) Microbiome disruption via ABX or LPS-ABX treatments significantly decreases species richness in the pup microbiome (Chao1 index) with respect to control or LPS groups, for both sexes. E) Disruption of the microbiome in the mother leads to significantly reduced alpha diversity (Shannon's H) in the male ABX pups, compared to male LPS or female ABX. F) Pups exposed to different gestational conditions show significantly different microbiome composition at postnatal day 40, as shown by the strong separation of samples and lack of overlap between different groups (p < 0.001). Group labels show the “average” position of each group (centroid), while coloured points show genera closest to the samples and groups where they were most abundant, with size of point indicating relative abundance. CCA: constrained correspondence analysis. N = 91 for all comparisons. ∗ = P=<0.05, ∗∗ = P=<0.01. (For interpretation of the references to colour in this figure legend, the reader is referred to the Web version of this article.)

High-throughput sequencing provided 16S rDNA profiles for 91 pup faecal samples (mean raw read-pairs per sample: 454K ± 129K, mean amplicon sequence variants (ASVs) per sample: 154K ± 38K merged read-pairs; mean unique ASVs per sample: 921 ± 170). While microbiome composition was largely similar across groups and gender (Fig. 8A–C), disruption caused by ABX exposure *in utero* was still evident in pups at P40. Species richness (Chao1, Fig. 8E) was significantly lower in all ABX and LPS/ABX groups (t = −3.5-2.7, p = 0.002–0.04), in addition to particularly low diversity in the male ABX group (Shannon's H; versus LPS: t = −2.5, p = 0.02; versus female ABX: t = 2.1, p = 0.035; Fig. 8D). In addition to a significant effect on individual samples (alpha diversity), treatment *in utero* also significantly changed microbial composition between treatment groups (beta diversity; PERMANOVA: F = 3.1, p < 0.001; Fig. 8F). In comparison, beta diversity was only weakly impacted by sex (PERMANOVA; F = 1.3, p = 0.09).

**Fig. 9:**
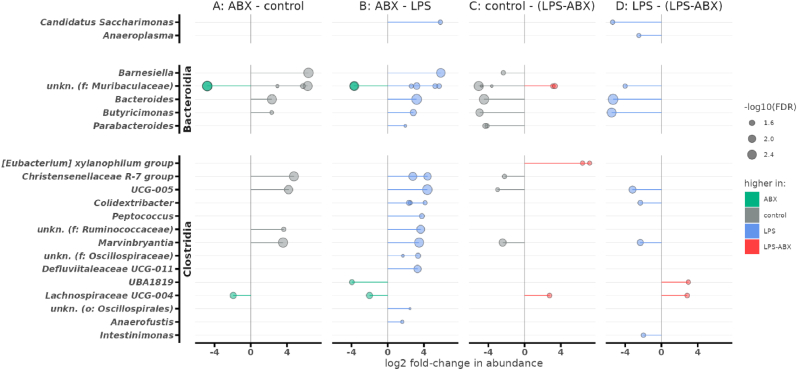
Many different populations of ASV are significantly affected by gestational microbiome disruption and inflammati*on atpostnatal day40.* Taxa which are significantly different between groups, while controlling for the effects of sex and litter. All features are significantly different below a false discovery rate (FDR) correction of 0.05, with size of point showing the FDR value on a -log_10_ scale (larger points are more significantly different). X axis shows the log_2_-fold change, i.e. how many doublings in abundance two groups differ by (L2FC 1 = log_2_(1) = 2 times more abundant, L2FC 3 = log_2_(3) = 8 times more abundant, etc.). In all cases, negative L2FC values indicate greater abundance in the left-hand side label. Abbreviations: f: family-level; o: order-level.

Maternal exposure to ABX/LPS-ABX during gestation was also associated with significant decreases in specific ASVs; mainly in [homo/heterofermenters and aceto/acido/butyrogens] from the taxonomic classes Bacteroidia and Clostridia (Fig. 9). The relative abundance of a number of uncharacterised taxa (*Muribaculaceae, Lachnospiraceae, UBA1819/Ruminococcaceae*) was increased in ABX/LPS-ABX exposure groups. Exposure to both LPS and ABX *in utero* was associated with increases in ASVs from the *Eubacterium xylanophilum* clade. Notably, there were no significant differences between the control and LPS groups, nor between the ABX and LPS-ABX groups.

## 4 Discussion

In this study, we noted significant deleterious effects on offspring weight, size, and neurobehavioural outcomes following exposure to two different prenatal stressors. LPS administration, simulating maternal bacterial infection, produced the most pronounced deficits in behaviour and neurochemical changes within the brain, along with various morphological changes. These offspring were significantly more anxious (Chen et al., 2023; Depino, 2015; Lin et al., 2012) and had an increase in repetitive behaviours (Fortunato et al., 2017; Kirsten and Bernardi, 2017) which corresponds with the previous literature. These shifts in behaviour were associated with changes seen in 5HT1a receptor expression in the prefrontal cortex, a key region in anxiety-related neural circuits. More discrete changes were noted following exposure to antibiotics *in utero*, where the main effect was evident in crown-rump length and placental weight. While cumulative effects were expected following exposure to both prenatal stressors, an interaction was only seen in the body weight of the embryos. This lack of a dual-hit effect of prenatal stressors on brain and behaviour is surprising. It was our expectation that the offspring exposed to both LPS/ABX would perform worse in the behavioural tests. Previous literature denotes the strong effect on neurodevelopmental sequalae that either stressor has on the offspring, hence a synergistic effect was hypothesised. Yet as the results show, this was not the case. It is difficult to state what may have blocked this effect, as this is the first time these exposures have been looked at in this context. One possible explanation for the lack of a dual-hit is a potential anti-inflammatory effect of the antibiotics. If the antibiotics were able to suppress the inflammation that LPS would be expected to produce, the two insults would essentially be counterintuitive. While not the same as the work performed in this study, no cumulative effects were seen in a model of neonatal LPS exposure and early adulthood antibiotic exposure (Tejkalová et al., 2023). Potentially the negative modulation of the maternal microbiome is preventing the LPS from having full effect, if certain pro-inflammatory species were depleted by the antibiotics the LPS may not be able to produce the same level of inflammation. Separately, it is likely that these two exposures work through separate mechanisms, MIA has been shown to influence neurodevelopment via the placenta (Woods et al., 2023), but antibiotic exposure may alter brain development through short-chain fatty acids for instance (Jin et al., 2022; Vuong et al., 2020). By working through two separate pathways, it is possible that this study may not capture any cumulative effects.

Exposure to LPS alone or both LPS/ABX were required to reduce crown-rump length, LPS/ABX to decrease body weight, but exposure to either stressor was sufficient placental weight. This strongly indicates that LPS exposure results in intrauterine growth restriction (IUGR) in the fetuses that may be causing small for gestational age offspring. Previous work has shown that LPS exposure leads to IUGR (Liu et al., 2014), and in humans both IUGR and being born small for gestational age have been linked to poorer neurodevelopmental outcomes (Sacchi et al., 2020). The growth restriction observed here may contribute to the behavioural changes seen later in life, but if this is the case, there is limited correlation with the severity of the outcomes. The placenta, which acts as the communicative interface between the mother and fetus for the duration of the pregnancy, is a vital organ necessary for sufficient oxygen and nutrient transport. Compromising the efficiency of the placenta is associated with numerous severe maternal complications, such as pre-eclampsia and placenta accreta spectrum, and may negatively influence fetal development (Cahill et al., 2018; Melchiorre et al., 2022). The reduction in placental weight seen in this study may be indicative of placental insufficiency and this in itself may lead to IUGR, fetal hypoxia, and preterm birth (Audette and Kingdom, 2018; Gagnon, 2003). It remains challenging to uncouple if placental insufficiency or IUGR has a greater impact on the negative offspring effects.

Ultrasonic vocalisation recordings have long been used as an insight into communicative behaviour between the pup-mother dyad and can elicit maternal approach. These calls can be interpreted as the pups’ affective state and can be informative about levels of postnatal stress. Various forms of prenatal stress have previously led to an increased number of calls emitted by the affected pups (Vitor-Vieira et al., 2024; Zimmerberg and Blaskey, 1998), but a decreased number of calls has also been noted (Baharnoori et al., 2012). However, no significant effects were seen in this study, suggesting that pups exposed to prenatal stressors did not exhibit heightened stress levels compared to their control counterparts. This result is surprising, as vocalisation changes were expected in the offspring. It is hard to deduce why no significance was reported in this study but there are a few possible suggestions. It may have been a better approach to record vocalisations across multiple timepoints, to get a wider sampling range in case any alterations to vocalisations may emerge earlier or later. This has been done with several previous studies and not testing at various early life timepoints may have masked any effects (Hermans et al., 2024; Lai et al., 2014). Building upon this, LPS and antibiotic exposure has been shown to influence brain development, they may not affect areas involved in vocal behaviour. The periaqueductal grey and amygdala are key regions involved, and while we only examined 5HT1aR expression in the amygdala, no changes were seen. It is very possible that the methods of prenatal stress do not evoke alterations in this behaviour.

The open field test revealed that offspring exposed to LPS showed greater anxiety-like behaviours, with an increased number of startled or freezing behaviours. In this, we found that male rats were driving the significant changes. While the increased number of freezing behaviours aligns with past literature, it is interesting that no changes were seen with the time spent in the centre of the arena, which would also be indicative of anxiety. This nuanced result may indicate that the anxiety seen from these offspring may not be baseline anxiety and may be more of fear avoidance. Both of these are regulated by partially distinct circuits, with overlap seen in the amygdala (Koutsikou et al., 2014; Yan et al., 2022), potentially explaining the differences seen here. The sex differences seen in this behaviour finding mimics what is normally found in the human population, where following prenatal stress, male offspring have 4- to 8- fold increased risk of developing various neurodevelopmental disorders (Bale, 2016). As previously mentioned, the increase in anxiety-like behaviours following prenatal stress is well-documented, though the precise mechanisms remain unclear. Potential contributors include disruptions of the serotonergic and noradrenergic systems, microglia activation, and alterations in the neuroanatomy (Quagliato et al., 2021).

The marble burying test indicated high levels of repetitive and stereotypical behaviours following LPS exposure, paralleling characteristics of obsessive-compulsive disorder (OCD) in humans. While OCD, a subcategory of anxiety, is typically less associated with neurodevelopment, it still has developmental underpinnings (Geschwind, 2011). Although prenatal stress typically affects males to a greater extent, this behavioural analysis revealed that females were more engaged in the repetitive behaviors. Yet with that being said, females are more likely to develop OCD, which aligns with the findings in this study (Fawcett et al., 2020). Typically, males and females present OCD symptoms differently with tics and social phobias deemed more male-associated symptoms, and compulsion deemed more female associated (Mathes et al., 2019). The parameters of this behavioural test associates closer with the expected presentation of female OCD. But, because of this, it is entirely possible that the male offspring also may display OCD-like behaviours but simply another test would be required to uncover them.

Maternal immune activation via LPS administration has been previously linked to social behaviour deficits, with the exposed offspring typically showing reduced interaction with conspecifics when compared to their control counterparts (Dutra et al., 2023; Lee et al., 2021), interpreted as an autism-like phenotype. However, this pattern was not replicated in the current study. In this study, no changes were seen in sociability or social novelty. Based on the current literature, our findings do not align with what has previously been shown. The discrepancies seen here could be stemmed back to various LPS dosing, as most studies administer one dose earlier or later than the first dose in this study (Dutra et al., 2023; Lee et al., 2021). Time of testing may also contribute, Dutra et al. noted opposing results on P28 and P60, showing that social behaviour may change over time. Perhaps our timepoint of P40 was too late, and any deficits caused by the LPS may have been compensated for. Not only is the lack of an LPS surprising, but direct antibiotic usage has previously be shown to affect social behaviours in rodents (Hayer et al., 2023). However, in terms of the offspring exposed to antibiotics *in utero*, the results are not as clear. Studies report mixed findings of decreased sociability but this finding has not been replicated in others (Hayer et al., 2023). Perhaps social behaviour is more difficult to characterise during this juvenile and adolescent period.

LPS exposure reduced the expression of 5HT1a receptors in the prefrontal cortex, and this change was driven by affected male offspring. The 5HT1a receptor is a well-documented inhibitory G-protein coupled serotonergic receptor that plays an important role in the presentation of various anxiety disorders. These heteroreceptors, often located on interneurons or pyramidal neurons, are implicated in the modulation of mood disorders (Akimova et al., 2009). Human imaging studies have revealed that these receptors are abundantly present in various regions of the brain, including the amygdala and prefrontal cortex (Garcia-Garcia et al., 2014). There was no significant difference in 5HT1a receptor expression in the amygdala, which is surprising considering the role of the amygdala in anxiety. The decrease in 5HT1a receptor expression seen in the prefrontal cortex was anticipated as the prefrontal cortex has long been involved in the neuronal signalling pathway associated with anxiety, with numerous projections to other important structures, such as the insula, locus coeruleus, and the stria terminalis (Bouras et al., 2023). Rodent studies have noted that 5HT1a receptors have a role in both somatosensory development, but are also necessary in early postnatal life to mediate the emergence of normal and expected anxiety-like behaviours (Garcia-Garcia et al., 2014). Mice with non-functional 5HT1a receptors have increased levels of anxiety and a critical developmental window of these receptors has been theorised (Garcia-Garcia et al., 2014). Depletion of these receptors, as seen here in this study, may be partly responsible for the increased anxiety-like behaviours seen in the male offspring, and the repetitive behaviours seen in the females. The serotonergic system begins formation between gestational day 11–12 (Lauder, 1990). Considering that one of the potential mechanisms behind maternal immune activation is disruption of serotonin neurotransmission (Quagliato et al., 2021), it is plausible that prenatal stress induced by LPS exposure may disrupt the correct formation of the serotonergic system due to exposure coinciding with the beginning of this critical development window. The reduction in 5HT1a receptor expression in the prefrontal cortex may explain why anxiety-like behaviours were seen males and not the females.

Differential behavioural profiles between the two sexes are well-documented, with numerous potential mechanisms proposed to account for this disparity. One major hypothesis involves the sexual dimorphism of the placenta. Studies have revealed that the male placenta responds and adapts to prenatal stressors differently in comparison to the female placenta (Bale, 2016; Pérez-Cerezales et al., 2018). There are a number of proposed molecular mechanisms underpinning these differences, including transcriptional sex differences in the placenta, glycogen availability affecting energy levels, and the ability to upregulate the protective enzyme 11β-hydroxysteroid dehydrogenase-2 when required, which converts cortisol to the inactive cortisone (O'Connell et al., 2013; Pérez-Cerezales et al., 2018; Wilcoxon et al., 2003). Furthermore, following early life stress, the levels of 17-β-hydroxysteroid dehydrogenase-3 have been shown to be reduced in the male placenta. 17-β-hydroxysteroid dehydrogenase-3 converts androstenedione to testosterone, leading to reduced levels of testosterone in the brain, and this demasculinised phenotype has been shown to have behavioural implications (Bale, 2016). Despite the numerous and plausible hypotheses, the main conclusion is that the male fetus is not as well-equipped to deal with stressors in comparison to the female fetus. This typically leads the male to be impacted more than the female, with greater risk of neurodevelopmental disorders in later life.

The subsequent manipulation of the maternal microbiome following LPS or ABX administration was found to leave a long-lasting imprint on the microbial composition of the offspring. The beta-diversity of the microbiome highlighted significant changes across all experimental groups, revealing that different groups are being driven by various bacteria with little-to-no overlap. Numerous populations of ASVs were also impacted by prenatal exposures, revealing variance between experimental groups in the abundance of different *Clostridia* and *Bacteroidia* species. Differences were particularly evident when examining species richness across the experimental groups, with LPS/ABX and ABX alone showing a significant reduction in the overall number of species present when compared to LPS and the control groups. Reduced species richness has been previously linked to disrupted behaviour, mainly anxiety and depression, and is also associated with increased inflammation (Bear et al., 2023; Kumar et al., 2023; Lee et al., 2018, p. 201). Yet, no behavioural alterations were found to be caused by *in utero* ABX exposure in this study, which suggests that the reduction in species was not substantial enough to impact behavioural changes. This is similar to the reduced levels of alpha-diversity seen in the ABX group when compared to LPS exposed offspring. Poor alpha-diversity is known to contribute to impaired gut and immune health, in addition to impacting behavioural outcomes (Lee et al., 2018; Li et al., 2023; Shevchenko et al., 2023; Wang et al., 2022). Interestingly, the reduction in alpha-diversity was only seen in the male offspring. There are established differences in male and female microbiomes, so this is not unexpected (Shobeiri et al., 2022). Yet the reason why this outcome was evident could be related to many potential mechanisms, such as the impact of hormones or sex-related behavioural differences (d’Afflitto et al., 2022). It is surprising however that LPS exposure is insufficient to induce its own changes to the gut microbiome, as this has been previously established to cause the induce an inflammatory-like phenotype in the human microbiome previously (An et al., 2022; Candelli et al., 2021).

Despite the lack of effect upon exposure to antibiotics *in utero* on the offspring, maternal immune activation led to an increase in anxiety-like and repetitive-like behaviours and altered the serotonergic system in the prefrontal cortex. While this study focused on serotonergic signalling, we acknowledge that both MIA and antibiotic exposure likely impacts immune pathways in both the brain and the gut of the offspring. It is possible that some of the behavioural outcomes seen in this study is in response to microglial activation or alterations in gut immune cells. Future studies should focus on neuroinflammation and possible ways to ameliorate the effects through positive modulation of the maternal microbiome. This could be achieved through administration of anti-inflammatory probiotics, such as various lactobacilli or bifidobacteria. To date, there have been few studies investigating the therapeutic benefit of probiotics following maternal inflammation, but future studies are warranted to help reduce offspring exposure to inflammation *in utero* and improve their neurodevelopmental outcomes.

## 5 Conclusion

In conclusion, we examined the effect of MIA and maternal microbial dysbiosis on offspring neurodevelopment via *in utero* exposure to LPS and/or an antibiotic cocktail. While minimal changes were seen due to antibiotic exposure, LPS exposure increased anxiety in males and repetitive-compulsive behaviour in females. These behavioural alterations are potentially mediated by the decreased expression of 5HT1a receptors in the prefrontal cortex. This potentially disrupts the correct formation of the serotonergic system during a critical period in early life. This work provides further evidence of how the sensitive developing brain may respond to prenatal stress.

## CRediT authorship contribution statement

**Clara Deady:** Writing – original draft, Methodology, Investigation, Formal analysis, Data curation. **Jamie FitzGerald:** Methodology, Formal analysis, Data curation. **Nirit Kara:** Methodology, Data curation. **Martina Mazzocchi:** Methodology, Data curation. **Adam O'Mahony:** Methodology, Data curation. **Mara Ioana Ionescu:** Writing – review & editing, Methodology, Data curation. **Ruth Shanahan:** Data curation. **Saoirse Kelly:** Data curation. **Fiona Crispie:** Writing – review & editing, Resources, Data curation. **Paul D. Cotter:** Writing – review & editing, Resources, Formal analysis, Data curation. **Fergus P. McCarthy:** Writing – review & editing, Visualization, Supervision, Project administration, Conceptualization. **Cathal McCarthy:** Writing – review & editing, Visualization, Supervision, Project administration, Conceptualization. **Gerard W. O'Keeffe:** Writing – review & editing, Visualization, Supervision, Project administration, Conceptualization. **Siobhain M. O'Mahony:** Writing – review & editing, Writing – original draft, Visualization, Supervision, Project administration, Funding acquisition, Conceptualization.

## Funding

This work was funded by the 10.13039/501100002081Irish Research Council (GOIPG/2022/426).

## Declaration of competing interest

The Authors have nothing to declare.

## Data Availability

Data will be made available on request.
